# Sex-specific effects of CD248 on metabolism and the adipose tissue lipidome

**DOI:** 10.1371/journal.pone.0284012

**Published:** 2023-04-28

**Authors:** Kieran Patrick, Xiang Tian, David Cartwright, Silke Heising, Matthew S. Glover, Ellie N. Northall, Lisa Cazares, Sonja Hess, David Baker, Christopher Church, Graeme Davies, Gareth Lavery, Amy J. Naylor

**Affiliations:** 1 Institute of Inflammation and Ageing, University of Birmingham, Birmingham, United Kingdom; 2 Dynamic Omics, Centre for Genomics Research, Discovery Sciences, Biopharmaceuticals R&D, AstraZeneca, Gaithersburg, MD, United States of America; 3 Institute of Metabolism and Systems Research, University of Birmingham, Birmingham, United Kingdom; 4 BioPharmaceuticals R&D, Cardiovascular, Renal and Metabolism (CVRM), Cambridge, United Kingdom; Vall d’Hebron Institut de Recerca, SPAIN

## Abstract

*Cd248* has recently been associated with adipose tissue physiology, demonstrated by reduced weight gain in high fat diet-fed mice with genetic deletion of *Cd248* relative to controls. Here we set out to determine the metabolic consequences of loss of *Cd248*. Strikingly, we find these to be sex specific; By subjecting *Cd248*^-/-^ and *Cd248*^+/+^ mice to a high fat diet and indirect calorimetry study, we identified that only male *Cd248*^-/-^ mice show reduced weight gain compared to littermate control wildtype mice. In addition, male (but not female) mice showed a lower respiratory exchange ratio on both chow and high fat diets, indicating a predisposition to metabolise lipid. Lipidomic studies on specific fat depots found reduced triglyceride and diglyceride deposition in male *Cd248*^-/-^ mice, and this was supported by reduced expression of lipogenic and adipogenic genes. Finally, metabolomic analysis of isolated, differentiated preadipocytes found alterations in metabolic pathways associated with lipid deposition in cells isolated from male, but not female, *Cd248*^-/-^ mice. Overall, our results highlight the importance of sex controls in animal studies and point to a role for *Cd248* in sex- and depot-specific regulation of lipid metabolism.

## Introduction

Adipose tissue is a highly sexually dimorphic tissue across many species. Lipid accumulation within separate anatomical regions is often part of secondary sexual characteristics [1], for example, male mice have a larger gonadal fat pad than females [2]. The lipid storage capacity of the perirenal fat pad is limited in male mice, and this results in metabolic syndrome during obesity models [3]. In humans, females are predisposed to accumulate fat in the lower body whilst males tend to accumulate fat in the abdomen, and this is linked to metabolic disease where the relatively greater levels of visceral fat observed in males are linked to a greater risk of developing type 2 diabetes [4].

Understanding the mechanisms that underlie sex-specific differences in lipid deposition, and the extent to which lipid accumulation in specific depots impacts whole-body metabolic activity, is a key area of investigation in the pursuit of developing treatments for metabolic disease. Sex differences in adipose tissue are driven in large part by differences in sex hormones [5, 6], and recently sex hormones have been shown to control the size of adipocyte progenitor pools [7]. Prior to sexual maturity, differential gene expression between sexes is driven by sex chromosome karyotype and developmental genes contributing to intrinsic sex differences and is independent of hormonal signals[1, 2, 8]. Thus, the effect of a particular gene’s expression within adipose tissue in males and females can be affected by both hormonal and cell-intrinsic gene expression differences.

*Cd248* (Endosialin, TEM1) is a transmembrane glycoprotein that is expressed almost exclusively on mesenchymal stem cell (MSC) lineage cells including osteoblasts [9], fibroblasts [10], and adipocytes [11]. *Cd248* expression is high during development and is then downregulated in normal adult tissue. In the adult, increased expression has been associated with pathological tissue remodelling and it is seen particularly highly expressed in cancer associated fibroblasts [12], in fibrotic kidney disease [13], in inflammation associated with arthritis [14], and in atherosclerotic plaques [15]. Recently, *Cd248* has been associated with lipid accumulation and metabolism [11, 16]. Specifically, *Cd248*^-/-^ mice are protected from obesity and glucose intolerance when fed a high fat diet [11] and the serum lipid profile of *Cd248*^-/-^ mice on a high fat diet is altered [16], suggesting differences in lipid synthesis pathways. Here, we demonstrate that reduced weight gain in *Cd248*^-/-^ mice on a high fat diet is, in fact, sex specific; with only males showing this phenotype. Additionally, we uncover depot-specific differences in fat pad lipid profiles, and site-specific alterations in gene expression.

## Materials and methods

### Chemicals and reagents

Isopropanol (Honeywell, catalog no. 34965-4X4L), Ethyl acetate (Millipore Sigma, catalog no. 103649), Methanol (Avantor, catalog no. 9830–3), Toluene (Sigma-Aldrich, catalog no. 650579), Acetonitrile (Fisher Chemical, catalog no. A955-4), Formic acid (Fisher Chemical, catalog no. A117-50) and Ammonium formate (Sigma-Aldrich, catalog no. 70221-100G-F).

### Mice

All animal experiments were performed in accordance with U.K. laws [Animal (Scientific Procedures) Act 1986] and with the approval of the Local Ethics Committees at the University of Birmingham. Every effort has been made to present all data in accordance with the ARRIVE guidelines (NC3Rs) [17]. The generation and genotyping of *Cd248*^-/-^ mice have been described previously [18]. Mice were subsequently backcrossed for >10 generations onto a C57BL/6J background and confirmed by SNP analysis (Transnetyx Inc, Cordova). Genotyping was carried by Transnetyx Inc, Cordova. Mice were maintained on a 12 h light/dark cycle at 21°C with *ad libitum* access to food and water. All mice were generated from CD248^+/-^ x CD248^+/-^ pairs. Mice were fed either a standard chow diet containing 10% kcal from fat, high in fibre containing complex carbohydrates (Special Diets Services U.K. RM3 (P) 801700), or a high fat diet containing 60% kcal from fat (Research Diets Inc, Rodent Diet With 60 kcal% Fat RD12492).

### High fat diet indirect calorimetry, weight gain and glucose tolerance study

Cohort breeding from *Cd248* heterozygote (*Cd248*^+/-^) male and female pairs resulted in 7x *Cd248*^+/+^ male, 10x *Cd248*^-/-^ male, 6x *Cd248*^+/+^ female and 7x *Cd248*^-/-^ female mice. Results from all animals are included in the results (heterozygote offspring were not included in any experiments). From weaning (week 4) until week 8 mice were fed standard chow. From 8 weeks of age mice were fed high fat diet for a further 16 weeks (until 23 weeks of age). At the end of the experiment, mice were anaesthetised with isoflurane and a bolus serum blood sample obtained through cardiac puncture. Fat pads (inguinal, scapular, perirenal, gonadal) were collected, snap frozen in liquid nitrogen and stored at -80˚C.

### CL316,243 injection study and primary cell isolation (preadipocyte extraction)

6–12 week old chow-fed mice were used. 15x *Cd248*^+/+^ male, 14x *Cd248*^-/-^ male, 7x *Cd248*^+/+^ female, 7x *Cd248*^-/-^ female. Results from all animals are included in the results.

### Indirect calorimetry

Indirect calorimetry to assess energy metabolism was performed using the PhenoMaster System (TSE systems, Bad Homburg, Germany). Between 6–8 weeks of age, baseline energy metabolism on chow diet was assessed. Subsequently, energy metabolism was assessed after 6 and 12 weeks of high fat diet feeding.

Oxygen consumption rate (VO_2_), carbon dioxide production (VCO_2_), heat production, respiratory exchange ratio (RER), and food and drink intake were each measured (method as previously described [19]) over a 72-hour time frame with only the last 48 hours included in the analysis. Briefly, mice were placed into the PhenoMaster System in litter-mate groups for 24 hours to acclimatise to the altered environment. Subsequently the mice were separated and housed individually for 72 hours to enable collection of accurate individual recordings, with the first 24 hours discounted whilst the mice acclimatized to single housing. At the end of each period of indirect calorimetry recording, mice were returned to their cage groups.

In CL316,243 stimulation studies, mice were placed into the PhenoMaster system in cage groups for 24 hours to acclimatise (as above) and subsequently single-housed for 3 hours to attain a baseline measurement. CL316,243 (1 mg/Kg body weight, PBS solution, intraperitoneal injection) was then administered, followed by a further 3 hours of measurement, after which mice were returned to their cage groups.

### Glucose tolerance test

Mice were fasted for a 6-hour period and a small incision made in the distal tail vein, from which baseline (and all subsequent) blood glucose measurements were taken. Mice were then given a glucose bolus (2 g/Kg body weight, water solution, intraperitoneal injection) and blood glucose measurements were taken using a Contour XT glucometer (Bayer) at 15, 30, 60 and 120 min.

### Preadipocyte extraction and cell culture

Preadipocytes were extracted from the stromal vascular fraction (SVF) of inguinal fat pads. Briefly, mice aged 8–12 weeks old on chow diet were culled by cervical dislocation and fat pads dissected. Fat pads were finely minced, and collagenase digested (37°C, 1 hour with constant agitation). The resulting cell suspension was filtered to remove debris (70 μm pore size) and pelleted (300*g*, 3 min.) to separate mature adipocytes (which remain in the supernatant) from pelleted preadipocytes. Mature adipocytes and supernatant were discarded, whilst the SVF was resuspended in DMEM/F12 (Gibco, 10% FCS, 2 mM L-GLN, 100 U/ml Pen-Strep) and plated onto tissue culture plastic. Preadipocytes were used in experiments at passage 0.

### Adipocyte differentiation protocol

Passage 0 preadipocytes were cultured to 80–100% confluency in DMEM/F12. Subsequently induction media was applied for 2 days and afterwards maintenance media applied for 5 days. Induction media: Insulin (bovine) 1 μg/ml Sigma (I6634), dexamethasone 0.25 μg/ml Sigma (D4902), IBMX 30 μg/ml Sigma (I5879), rosiglitazone 1 μM (Cayman #71740). Maintenance media: Insulin (bovine) 1 μg/ml Sigma (I6634). At the end of the protocol cells were used for RNA or metabolite extraction.

### RNA extraction and cDNA synthesis

Cells or tissues were suspended in RLT buffer containing 1% w/v β-mercaptoethanol. Cells were lysed by agitation and repeated aspiration; tissue was lysed using the TissueLyser Sample Disrupter system (QIAGEN). Lysates were homogenised by passing through QIAshredder (QIAGEN) with centrifugation (2 min, 18,000 rcf). Samples were then taken through RNA Mini Kit protocol (QIAGEN) as per the manufacturer’s instructions. DNase digestion was performed on column. RNA concentration was determined by nanodrop (ThermoFisher). cDNA synthesis was performed on 500 ng of RNA in a 20 μL reaction using the High-Capacity cDNA Reverse Transcription Kit (Applied Biosystems) as per the manufacturer’s instructions.

### Gene expression analysis

Preamplification of cDNA was performed: 8 μL total reaction volume comprising 4 μL Taqman Preamp Mastermix, 2 μL Custom Preamp pool of primers (ThermoFisher, Waltham, USA) and 2 μL cDNA. Thermocycler protocol: 95°C for 10 mins, 14 cycles of 95°C for 15 seconds and 60°C for 4 mins. Samples were diluted 1:10 with nuclease free water and 3 μL sample was added into an OpenArray 384 well sample plate with 3 μL OpenArray Real Time PCR Master Mix. Samples were loaded by OpenArray Accufill System onto Custom Taqman OpenArray plates (ThermoFisher, Waltham, USA) containing 56 Taqman gene expression assays. Plates were sealed according to the manufacturer’s instructions and run on a QuantStudio 12K Flex Real-Time PCR System (ThermoFisher, Waltham, USA). Gene expression results were normalised to the reference genes PPIA, RPLP0, B2M and HPRT. Differentially expressed genes were defined as having a > 1.5-fold change from control and p < 0.05 (t-test). Details of the targets within the panel are in **S5 Table**.

### Luminex cytokine & chemokine 36-Plex mouse ProcartaPlex™ panel 1A

Frozen serum samples from high fat diet mice were thawed on ice and run through the Luminex assay according to the manufacturer’s instructions. Briefly, samples were diluted according to the kit supplied protocol and added to 96 well plate alongside protein standards. Primary antibody conjugated magnetic beads were incubated in the samples for 2 hours. The beads were then washed. Next detection antibodies were added and incubated for 30 min. Beads were washed and streptavidin-PE was added and incubated for 30 min. Beads were washed and resuspended in reading buffer. Data were acquired on a Luminex system. Details of the targets within the panel are in **S5 Table**.

### Lipid extraction adipose tissue

Extractions were performed using the following extraction solvent mixture IPA:H_2_O:EtOAc (30:10:60, v:v:v). 10 mg samples of adipose tissue were collected in 2.0 mL snap-top microcentrifuge tubes containing four 2.8 mm ceramic beads (VWR 215913). Samples were extracted in 1:10 volume of extraction solvent (10 mg tissue: 100 μL solvent mixture). Samples were homogenised with Tissue-Lyser (QIAGEN) for 2 min. at 30 Hz, then centrifuged to separate the organic phase from cellular debris (15 min. 18,000 *g*, 20°C). 10 μL of the organic upper phase were transferred to fresh microcentrifuge tube. Samples were then dried in a SpeedVac (Thermo) without heat and stored at -80°C.

Lipids were reconstituted in 400 μL MeOH:toluene (90:10 v:v) with 0.5 μg/ml Equisplash (Avanti) as internal standard, mixed for 1 min. at 1500 rpm (Eppendorf ThermoMixer C) followed by a sonication for 2 min. Samples were mixed for another 1 min. at 1500 rpm before centrifugation for 5 min. at 16,000 *g*, 20°C. 50 μL of the supernatant from each sample were transferred to glass HPLC vials and 5 μL of each individual sample were combined to make the pooled QC sample [20]. Samples were randomized before analysing by LC-MS, with a QC run after every 10 study samples.

### Sample extraction of differentiated adipocytes for metabolomics

Extractions were performed using the solvent mixture 50% ACN, 50% H_2_O, 0.1% formic acid, and a 1μM isotopic labelled amino acid mixture as internal standards (Cambridge Isotope Laboratories Inc. MA, USA). Confluent wells of cells were aspirated completely of media and washed in ice cold PBS. Plates were placed onto dry ice and 1ml of extraction solvent was added to each well. Next the plates were incubated at -80°C for 10 min. Cells were thawed, scraped, and collected into snap-top microcentrifuge tubes, vortexed, then centrifuged at 16,000*g* at 4°C. 900 μL of supernatant was collected into fresh snap-top microcentrifuge tubes and evaporated to dryness in a Speedvac.

Metabolites were reconstituted in 60 μL ACN:H_2_0 (3:1 v:v), then mixed for 1 min. at 1500 rpm (Eppendorf ThermoMixer C) followed by sonication for 2 min. Samples were mixed for another 1 min. at 1500 rpm before centrifugation (5 minutes at 16,000 *g*, 20°C). 50 μL supernatant from each sample was transferred to a glass HPLC vial (Waters) and analysed by LC-MS. 5 μL of each individual sample were combined to make a pooled QC. Samples were randomised before analysis by LC-MS with a QC run after every 6 study samples.

### LC-MS methods for lipidomics

LC-MS was performed on a Vanquish UHPLC (Thermo Scientific) coupled with Thermo Scientific Orbitrap ID-X Tribrid Mass Spectrometer. RP-LC was performed on a Vanquish UHPLC (Thermo Scientific), using a Waters ACQUITY UPLC CSH C18 column (1.7 μm, 2.1 mm × 100 mm, 130 Å) (P/N-186005297) with a sample injection volume of 1 μL. Mobile phase A consisted of 40% H_2_O and 60% ACN with 0.1% formic acid and 10 mM ammonium formate and mobile phase B was formed from 10% ACN and 90% IPA with 0.1% formic acid and 10 mM ammonium formate delivered at a constant flow rate of 0.6 mL/min. The gradient began with 15% mobile phase B and linearly increased to 30% from 0 to 2 min., then to 48% from 2 to 2.5 min., followed by a continuous increase to 82% from 2.5 to 11 min., finally rising to 99% from 11 to 11.5. After maintaining mobile phase B at 99% for 0.5 min. the percentage was reduced to 15% from 12 to 12.1 min. To re-equilibrate the column, 15% mobile phase B was used from 12.1 to 15 min. The temperature of the column oven was maintained at 65°C.

Mass spectrometry was performed on a Thermo Scientific Orbitrap ID-X Tribrid Mass Spectrometer, equipped with the EASY-IC internal calibration source, a divert/inject valve, and the Thermo Scientific OptaMax NG API source. MS1 analyses were conducted in positive ion mode using the following parameters (Voltage: 3500V positive ESI, Sheath gas: 50, Aux gas: 10, Sweep gas: 1, Ion transfer tube: 325°C, Vaporizer: 300°C, Resolution: 60k, Scan range: 60–900 *m*/*z*, AGC target: standard, microscans: 1). MS2 spectra of the pooled sample were acquired by AcquireX DeepScan iterative data-dependent acquisition workflow (ThermoFisher) method with the following parameters (Quad isolation: 1 m/z, Resolution: 7.5k, Scan Range: 200–1200, AGC Target: Standard, microscans: 3).

### LC and MS methods for metabolomics

LC-MS was performed on a Vanquish UHPLC (Thermo Scientific) coupled with Thermo Scientific Orbitrap ID-X Tribrid Mass Spectrometer. HILIC-LC was performed on an Vanquish UHPLC (Thermo Scientific), using a Waters ACQUITY UPLC BEH Amide HILIC column (1.7 μm, 2.1 mm × 150 mm, 130 Å) (P/N– 186004802) with a sample injection volume of 1 μL. The mobile phase A consisted of 100% H_2_O with 0.125% formic acid and 10 mM ammonium formate) and mobile phase B was 95% ACN and 5% H_2_O with 0.125% formic acid and 10 mM ammonium formate delivered at a constant flow rate of 0.4 mL/min. The gradient began with 100% mobile phase B for 2 mins and linearly decreased to 70% from 2 to 7.7 min. Then, dropped to 40% from 7.7 to 9.5 mins. After that, it continuously decreased to 30% from 9.5 to 10.25 min, then increased to 100% B from 10.25 to 12.75. To re-equilibrate the column, 100% mobile phase B was maintained from 12.75 to 16.75 min. The temperature of the column oven was set at 45°C.

Mass spectrometry was performed on a Thermo Scientific Orbitrap ID-X Tribrid Mass Spectrometer, equipped with the EASY-IC internal calibration source, a divert/inject valve, and the Thermo Scientific OptaMax NG API source. MS1 analyses were conducted using a polarity switching approach, where the Orbitrap ID-X MS was operated by alternating between positive and negative ESI modes using the following parameters (Voltage: 3500V positive ESI and 3000V negative ESI, Sheath gas: 50, Aux gas: 10, Sweep gas: 1, Ion transfer tube: 325°C, Vaporizer: 300°C, Resolution: 30k, Scan range: 60–900 m/z, AGC target: standard, Microscans: 1). MS2 spectra of the pooled sample were acquired by ddMS2 (deep scan) method with the following parameters (Quad isolation: 1 m/z, Resolution: 7.5k, Scan Range: 60–900, AGC Target: Standard, Microscans: 1).

### Data and statistical analysis

Lipidomics and metabolomics data analysis, including peak picking, alignment, smoothing, and annotation was performed using MSDial software. Feature annotation followed the Metabolomics Standards Initiative (MSI) levels [21]. The lipidomics data were normalized to the internal standard, EquiSPLASH (Avanti, Birmingham, AL, USA, SKU: 330731). The metabolomics data were normalized to total ion current (TIC). To remove the background noise, S/N threshold was set to 3. In addition, the lipids, or metabolites with relative standard deviation (RSD) higher than 30% in the pooled QC samples were also removed. The data were analysed in R using both in-house and MetaboAnalyst scripts, followed by analysis in GraphPad Prism software. Significance was calculated either by t-test when only one comparison was made, or by ANOVA where multiple comparisons were made. For gene expression data, differences were considered significant if t-test ≤ 0.05 along with fold change ≥ 1.5. In the case of the indirect calorimetry data, where ANCOVA analysis was required to determine if the body weight covariable was determining RER, this was carried out using SPSS software ANCOVA tool. Data are presented as either ± standard deviation (weight gain, food intake, average RER, gene expression) or ± standard error of the mean (CL316,243 response, glucose tolerance test). Metabolite identifications were made according to the recommendations contained in the Metabolomics Society Initiative (MSI) as defined by Sumner et al (2007)[21]. All metabolomics data has been uploaded to the Metabolights repository, ID MTBLS6615:

https://www.ebi.ac.uk/metabolights/MTBLS6615/descriptors.

## Results

**Fig 1:**
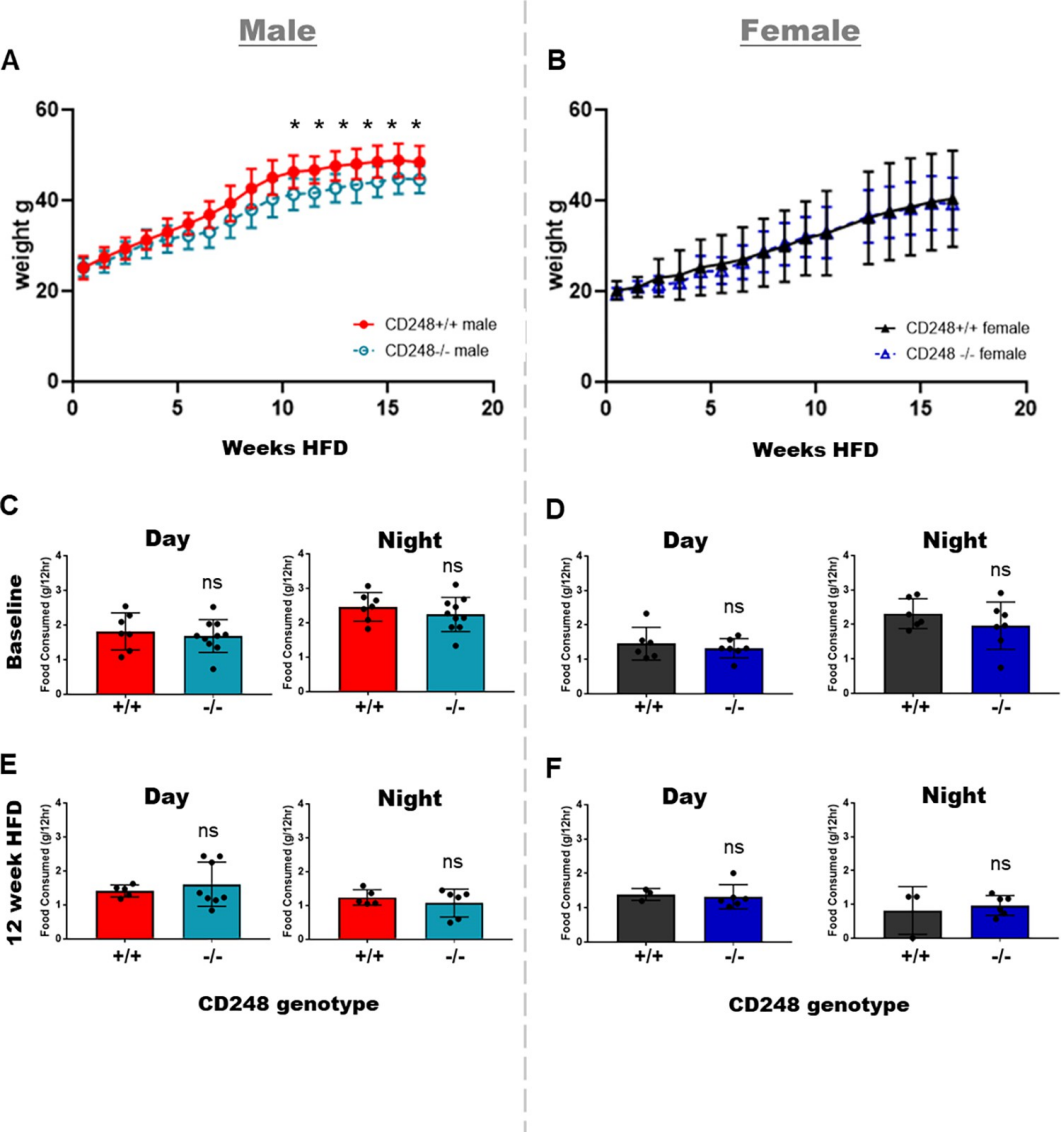
Weight gain ±SD over 16 weeks of high fat diet in **(A)** males and **(B)** females. Food intake ±SD at baseline **(C)** day and **(D)** night. Food intake over 12 hours after 12 weeks of high fat diet during the **(E)** day and **(F)** night. Body weight data over time were analysed for significant differences by t-test. Food intake was analysed by t-test. In all graphs: Cd248^+/+^ males (red), Cd248^-/-^ males (cyan), Cd248^+/+^ females (black), Cd248^-/-^ females (blue). Significant * p ≤ 0.05.

**Fig 2:**
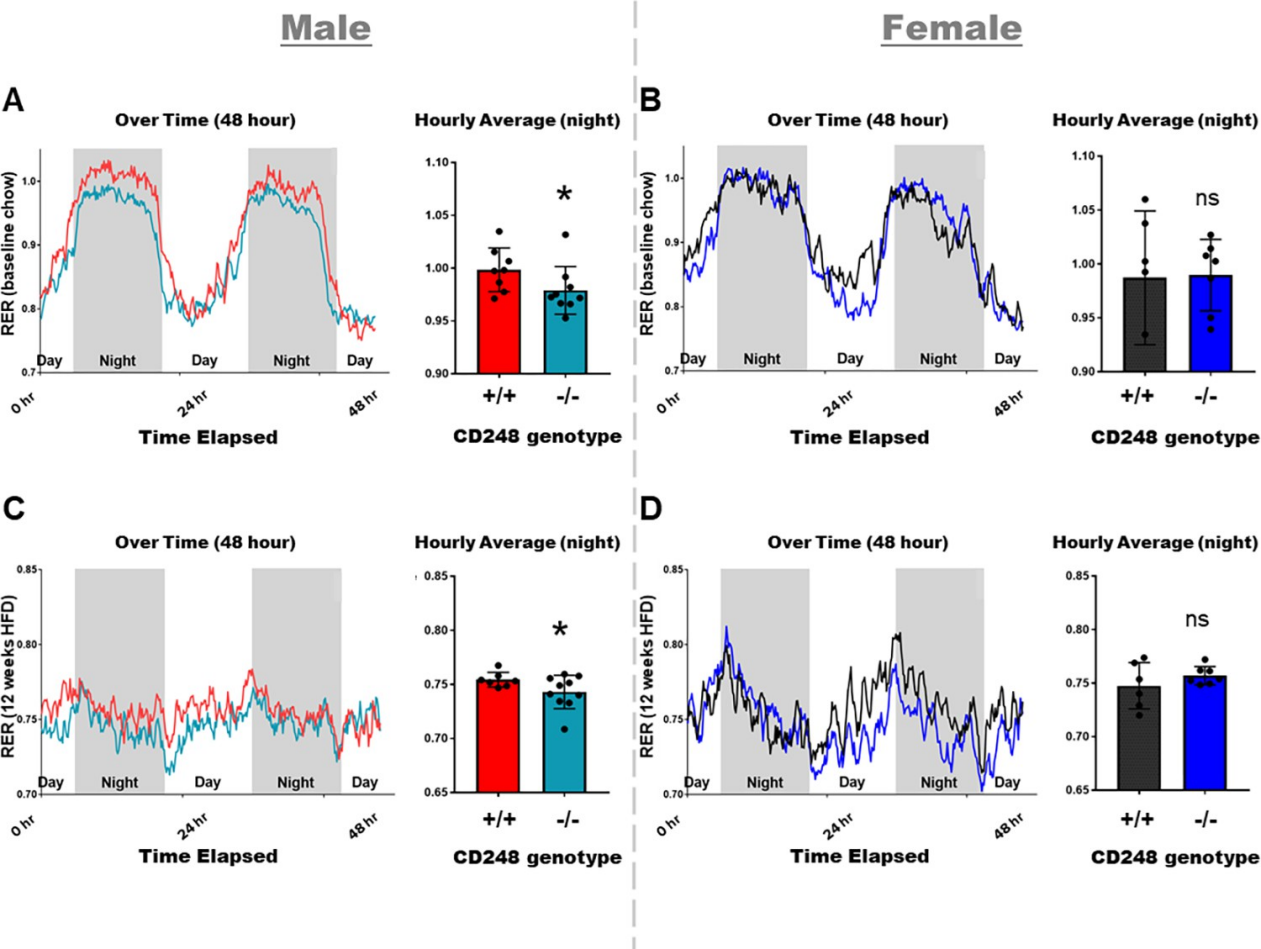
Respiratory exchange ratio (RER) as calculated from indirect calorimetry measurements over 48 hours: Chow-fed **(A)** males and **(B)** females; HFD-fed **(C)** males and **(D)** females. Line graphs indicate hourly averages over time whilst bar graphs show overall averages at night ± SD. Body weight data over time were analysed for significant differences by t-test. RER data were analysed for significance by ANCOVA test to determine if the body weight covariable is responsible for the significant changes in RER. In all graphs: Cd248^+/+^ males (red), Cd248^-/-^ males (cyan), Cd248^+/+^ females (black), Cd248^-/-^ females (blue). Significant * p ≤ 0.05.

To determine if genetic deletion of *Cd248* confers protection against adiposity via changes to energy metabolism, mice were subjected to a high fat diet and weighed weekly for 16 weeks in parallel with a series of indirect calorimetry measurements. A sex-specific difference in weight gain was observed: Male *Cd248*^-/-^ mice gained significantly less weight compared to littermate controls, whilst female *Cd248*^-/-^ mice showed equal weight gain to littermate controls (**Fig 1A & 1B**). No differences in food consumption were detected either on chow or on high fat diet for either sex regardless of genotype (**Fig 1C -** **1F**). To detect whether alterations in energy metabolism and/or fuel utilisation between *Cd248*^-/-^ mice and littermate controls was the cause of the reduced weight gain phenotype, indirect calorimetry data was analysed for significance by ANCOVA. We found sex-specific differences in RER: male *Cd248*^-/-^ mice showed significantly lower RER during the night period, both at baseline on chow diet and at 12 weeks of high fat diet (**Fig 2A & 2C**), whilst no genotype-specific differences were detected between females (**Fig 2B & 2D**). A lower RER implies that the *Cd248*^-/-^ male mice were oxidising more lipids than their wildtype littermates.

**Fig 3:**
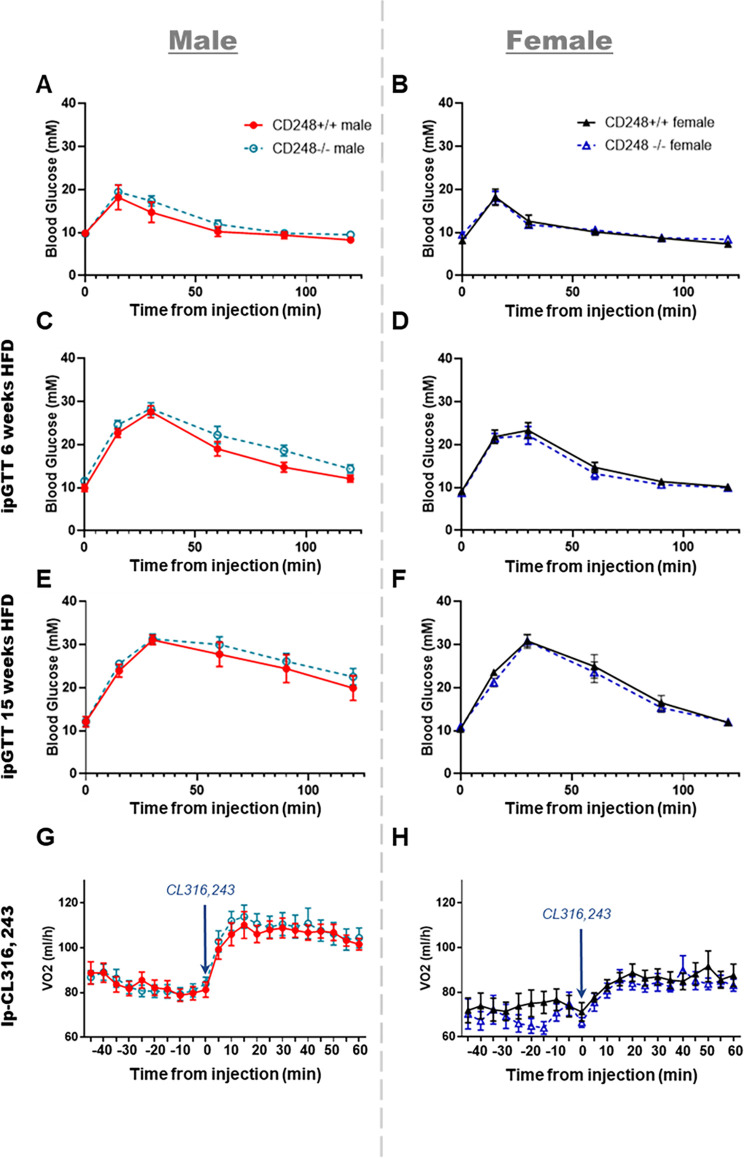
Glucose tolerance test (GTT) was performed at baseline in **(A)** males and **(B)** females (6–8 weeks old mice) and following high fat diet-feeding for 6 weeks **(C)** males and **(D)** females, then 15 weeks **(E)** males and **(F)** females. Response to CL316,243 injection was measured as average VO2 over time in **(G)** males and **(H)** females. In all graphs: Cd248^+/+^ males (red), Cd248^-/-^ males (cyan), Cd248^+/+^ females (black), Cd248^-/-^ females (blue). Data were analysed for significant differences by ANOVA. No significant differences were found. All error bars ± SEM.

As *Cd248* has been associated with inflammatory states in arthritis models [14], and given that inflammation can alter metabolic rate, one possibility behind the reduced weight gain was that it was due to an altered inflammatory state of the *Cd248*^-/-^ males following 15 weeks HFD. Thus, quantification of 36 cytokines and chemokines (Luminex) known to be involved with inflammation was performed in the serum of the mice following 15 weeks of high fat diet (**S1 Fig**). No differences in these markers of inflammation were identified between *Cd248*^+/+^ and *Cd248*^-/-^ in either sex. To assess whether loss of Cd248 was protective against metabolic syndrome, glucose tolerance tests (GTT) were carried out at baseline (age 6–8 weeks), and 6 weeks, 12 weeks, and 15 weeks of high fat diet-feeding. Despite reduced weight gain, *Cd248*^-/-^ males showed no difference in their response to GTT at any of the timepoints measured (**Fig 3A, 3C and 3E**). Females also showed no difference (**Fig 3B, 3D and 3F**). The reduced weight gain observed led us to hypothesise that male *Cd248*^-/-^ mice had higher levels of adipocyte browning. To test this hypothesis, mice were stimulated with the β-adrenergic agonist CL316,243 whilst VO2 was recorded in the PhenoMaster system. No significant differences were found. (**Fig 3G & 3H**).

**Fig 4:**
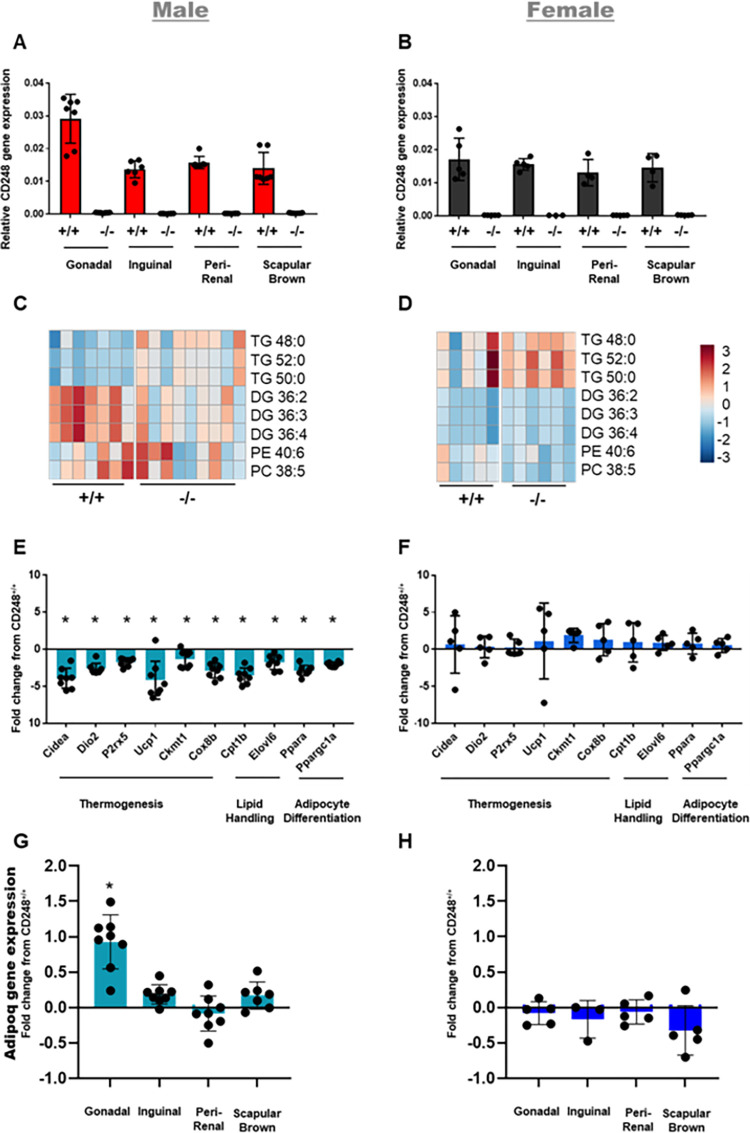
Gene expression and lipidomic results from fat pads following high fat diet feeding for 16 weeks. CD248 expression analysis in each fat pad confirmed loss of CD248 in Cd248^-/-^ tissues from **(A)** males **(B)** females. Heatmap showing z—scores (scale bar from blue = -3 to red = +3 shown—far right) of significantly altered lipids in **(C)** male Cd248^-/-^ perirenal fat pad compared to Cd248^+/+^ with **(D)** female shown for comparison where no significant differences were detected. Lipid notation follows the nomenclature described in Liebsch *et al*. 2013 [22]. Detail of the MSI identification level for each of the lipid species is given in **S1 Table**. Gene expression array showing significantly altered genes from **(E)** male Cd248^-/-^ perirenal fat pad, with **(F)** female shown for comparison where no significant differences were detected. Adipoq expression data for all four fat depots in (**G**) male and (**H**) female mice are also shown. Results analysed for significant differences by t-test. Considered significant (*) if t-test yielded p ≤ 0.05 and fold change or z score from control ≥ 1.5. All error bars ± SD.

Following 16 weeks of high fat diet-feeding, fat depots were assessed by mass spectrometry (by LC/MS) and gene expression analysis (**Fig 4**). *Cd248* gene expression was detected at similar levels in the fat pads of wildtype animals, regardless of sex and was undetectable in all adipose tissues from *Cd248*^-/-^ animals (**Fig 4A and 4B**). Lipidomic analysis of the scapular BAT, inguinal WAT, gonadal WAT and perirenal WAT identified sex-specific differences in lipid classes (**S2 Fig**): In the perirenal pads, *Cd248*^-/-^ males had lower amounts of diglycerides compared to *Cd248*^+/+^ males (**Fig 4C**). No significant differences were observed in fat pads from female mice (**Fig 4D**). RNA expression analysis was performed using a custom chip of 56 Taqman gene expression assays (comprising 52 target genes and 4 reference/housekeeping genes) against adipocyte associated genes, chosen to cover a range of different pathways and processes including adipocyte browning, lipid storage, mitochondrial function, and lipid metabolism. This analysis also identified sex-specific and depot-specific gene expression changes in male, but not female mice; notably the perirenal WAT of male *Cd248*^-/-^ mice showed reduced expression of 10 genes involved in thermogenesis (*Cidea*, *P2rx5*, *Cox8b*, *Ckmt2*, *UCP1*, *Dio2*), lipid handling (*Cpt1b*, *Elovl6*) or adipocyte differentiation (*Ppara*, *Ppargc1a*) relative to *Cd248*^+/+^ controls (**Fig 4E**), whilst *Adipoq* gene expression was increased in the gonadal fat pad only (**Fig 4G**). These changes were not identified in female mice (**Fig 4F and 4H**). Inguinal and scapular fat pads showed fewer differences compared to perirenal and gonadal fat pads (**S3 Fig**) indicating that visceral adipose tissue depots were most affected by genetic deletion of *Cd248*.

**Fig 5:**
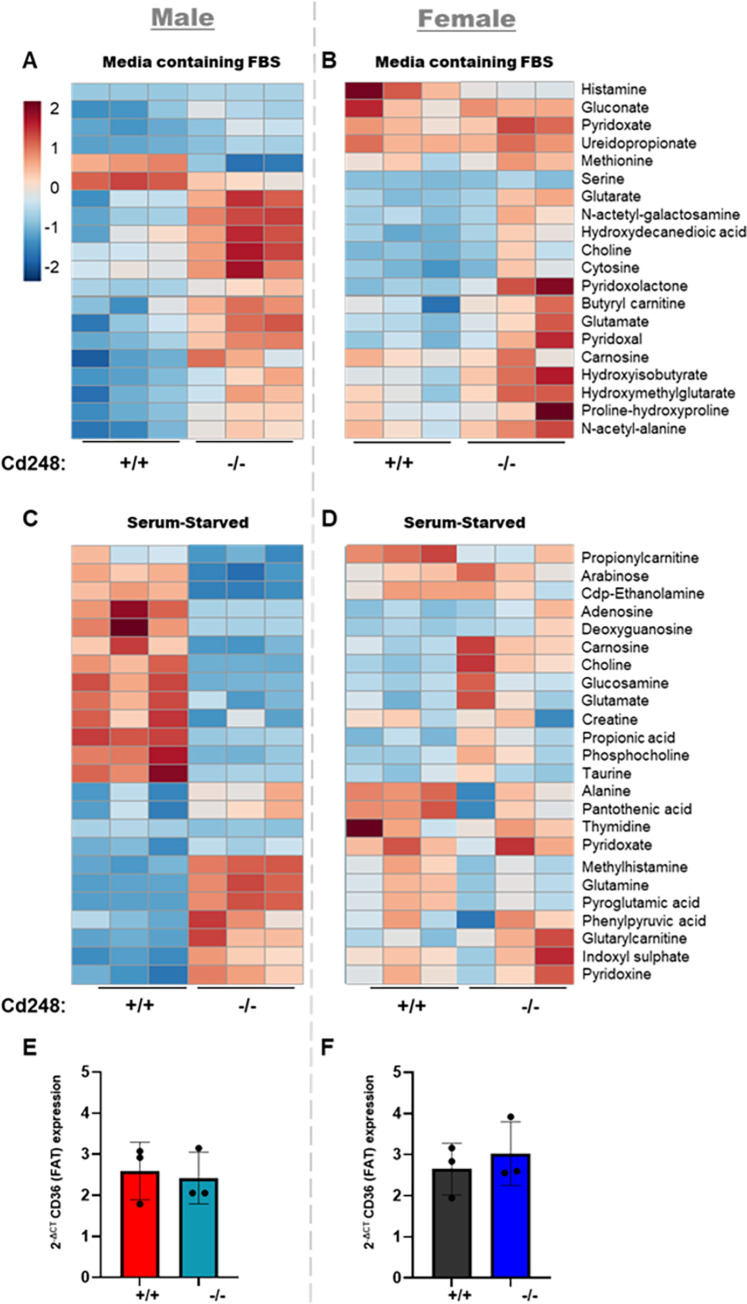
Results from differentiated preadipocytes. Heat map showing z–scores for metabolites identified as significantly altered in cells isolated from male mice. Cd248^+/+^ and Cd248^-/-^ differentiated preadipocytes from chow fed (**A)** males and (**B)** females were cultured in 10% FBS media (Media containing FBS) or following 2 hours of serum starved conditions: **(C)** males and **(D)** females. Scale bar from blue = -2 to red = +2 (shown top left). Analysis for significant differences by t-test. Considered significant if t-test yielded p ≤ 0.05 and z—score from control ≥ 1.5. Detail of the MSI identification level for each of the metabolite species is given in **S2** and **S3 Tables** for culture in media containing FBS and serum starved conditions, respectively. Expression levels of adipocyte differentiation marker, CD36 (normalised to housekeeping gene) are shown for (**E**) male and (**F**) female-derived cells. No significant difference was detected.

Given the altered weight gain and energy metabolism in *Cd248*^-/-^ males, alongside the differences in lipid profile and gene expression in *Cd248*^-/-^ male adipose tissue, we hypothesised that altered metabolic pathway(s) were intrinsic to *Cd248*^-/-^ male adipocytes. To test this hypothesis, we performed metabolomic analysis by LC-MS on primary cultured differentiated preadipocytes extracted from the inguinal fat pad of male and female *Cd248*^+/+^ and *Cd248*^-/-^ mice (**Fig 5**). Analysis was performed on cells cultured under normal (10% FBS) serum conditions (**Fig 5A an d 5B**) and following 2 hours serum starvation (**Fig 5C and 5D**). Several metabolites were found to be significantly different between *Cd248*^+/+^ and *Cd248*^-/-^. Including those involved in vitamin B6 metabolism (pyridoxal, pyridoxalacetone, pyridoxate) or glutamine metabolism (glutarate, glutamate). More acute differences were observed between *Cd248*^*+/+*^ and *Cd248*^*-/-*^ in the male derived cells under serum starvation compared to the female. Expression of adipocyte differentiation marker, CD36 was equivalent across cells differentiated from all sexes and genotypes (**Fig 5E and 5F**).

## Discussion

The reduced weight gain phenotype seen in *Cd248*^-/-^ males fed a high fat diet, whilst relatively mild, is consistent and robust, having been previously reported by a separate research group using a *Cd248*^-/-^ mouse on a different background strain [11]. No such differences were seen in weight gain for *Cd248*^*-*/-^ fed a chow diet [16]. Additionally, no differences were identified in circulating lipid levels or lipid accumulation in *Cd248*^-/-^ mice fed chow diet, whilst these were identified on HFD [16]. These previous findings suggest that HFD stress is required to induce a metabolic syndrome phenotype in the absence of *Cd248*. Petrus et al (2019) also reported protection from glucose intolerance in *Cd248*^-/-^ fed a high fat diet, however, this phenotype was not replicated in our study [11]. This disparity could be due to the variance in mouse strain or another difference in animal housing or conditions of which we are not aware. Indeed, mouse strain and sex are critical variables to consider in the interpretation of metabolic phenotypes identified from genetically modified mice. Ingvorsen et al (2017) previously assessed the effect of sex on response to HFD in a large sample-set (N = 659 female, 660 male) of C57BL/6N mice, and demonstrated that male mice are more sensitive to high-fat diet-induced obesity and to glucometabolic changes than female mice of the same strain [23]. In another large study (>300 mice), Wang et al (2006) explored the influence of sex on inheritance of quantitative trait loci (QTL) and their subsequent influence on obesity in the APOE knockout mouse, a strain used widely in obesity and metabolic syndrome studies [8]. They identified a “remarkably high degree of sex-dependence on both the cis and trans regulation of gene expression” [8]. We have controlled against the risk of genetic drift by comparing mice from cohorts of heterozygous pairings to ensure analysis of littermate controls throughout this study. Expression of CD248 between male and female wildtype mice is equivalent in all fat pads except the gonadal, where expression is higher in males. Given that significant differences in genes involved in thermogenesis, lipid handling and adipocyte differentiation were seen only in the male perirenal fat pad, we do not conclude that sex differences in CD248 gene expression are the primary cause of this phenotype.

By performing experiments on male and female wildtype mice in parallel, we can see that female mice showed a metabolic response to HFD in this study, as seen by alterations in RER and glucose tolerance, although weight gain was more variable in the female cohort.

To understand the cause of the reduced weight gain identified specifically in male *Cd248*^-/-^ mice, we performed indirect calorimetry, which identified a lower RER in *Cd248*^*-/-*^ males relative to littermate *Cd248*^*+/+*^, indicative of increased lipid oxidation. We investigated whether the reduced weight gain in *Cd248*^*-/-*^ males was due to either inflammation or adipocyte browning. However, we found no differences in inflammatory cytokines in the serum of *Cd248*^*-/-*^ mice on high fat diet. We also found no difference in expression of adipocyte browning-associated genes and no difference in the response to beta–adrenergic stimulation between *Cd248*^-/-^ mice and littermate controls. These data do not suggest that an underlying pro-inflammatory or adipocyte browning phenotype is the primary cause of the increased lipid oxidation. Of note, there is a lack of consensus in the published data concerning the gene and protein expression of CD248 in brown adipose tissue. Here, in the mouse, we detected similar levels of mRNA in white (gonadal, inguinal, and peri-renal) depots as compared to scapular brown fat, confirming the findings of Roh et al. (2018) but at odds with those of Petrus et al. (2019) who did not detect Cd248 in BAT [11, 24]. At the protein level, Muller et al. (2016) identified CD248 in human BAT whilst Li et al. (2020) did not detect it in murine BAT [25, 26]. The phenotypes we have detected in the Cd248^-/-^ mouse are all seen in the WAT compartment, where Cd248 expression is not disputed, however we highlight this uncertainty to the reader for completeness and consideration when interpreting the findings.

Lipidomics uncovered a difference in lipid profile in the male *Cd248*^*-/-*^ perirenal fat pad. Diglycerides alongside phosphatidylethanolamine and phosphocholine were found to be altered. This observation is noteworthy as these lipid species are utilised in synthesis of the cell membrane, and this corroborates the possible differences in the Lands Cycle observed by us previously in plasma [16]. Differences in the lipids detected in fat pads imply that *Cd248* has a role in localised fat deposition. Although the mechanism behind this is unclear, it is supported by gene expression changes observed in specific fat depots. In perirenal WAT, we find lower expression of transporter *Cpt1b*, which imports fatty acids to mitochondria [27]; reduced expression of enzyme *Elovl6*, which is required for fatty acid polymerisation [28, 29]; lower levels of *Cidea*, a transcription factor known to regulate lipolysis and energy expenditure [30] and downregulation of *Dio2*, which is associated with adipocyte browning [31] and has also shown a sex-specific phenotype [32]. Downregulation of *Ppara* was also observed in *Cd248*^-/-^ mice, which is a known regulator of adipocyte differentiation and lipid metabolism [33]. In gonadal WAT there is significantly higher expression of the adipokine Adiponectin (Adipoq), which is known to promote fatty acid oxidation and impair adipocyte differentiation in mice [34, 35].

The metabolites found to be altered by LC-MS include those involved in vitamin B6 metabolism (pyridoxal, pyridoxalacetone, pyridoxate). Vitamin B6 has been associated with lipid deposition since the 1970s [36] and more recently expression of a kinase in this pathway was found to control adipogenesis and insulin signalling [37]. Metabolites from glutamate metabolism are also altered (glutarate, glutamate, glutamine). Glutamine has recently been demonstrated to correlate with inflammation of adipose tissue in obesity [38]. Glutarylcarnitine was found to be elevated in cells derived from male *Cd248*^-/-^ mice, when cultured in the absence of serum, whilst O-acetylcarnitine was found to be elevated in normal conditions. These findings correlate with the observation that expression of *Cpt1b* was downregulated in male *Cd248*^-/-^ adipose tissue, given that this transporter is required for uptake of carnitines into mitochondria for beta-oxidation.

The differences identified in cells and tissues from male *Cd248*^-/-^ mice were not evident in female mice. Similarly, no phenotypic differences were observed in female *Cd248*^-/-^ compared to their *Cd248*^+/+^ littermates. These experimental results demonstrate a sex-specific role for *Cd248* in lipid deposition in mice, adding to the body of knowledge of sex differences in adipose tissue metabolism. We cannot exclude the influence of sex hormones (including their potentially long-lasting effects in our primary cell culture findings) or the potential role of sex-linked inheritance of CD248-interacting genes in the causation of the male-specific phenotype observed on genetic deletion of CD248. Petrus et al (2019) previously demonstrated a reduction in CD248 expression in human females following post-bariatric surgery [11]. Whilst a similar male cohort was not available for comparison in this case, and correlation does not necessarily imply causation, these results, in the light of our findings, reinforce the importance of using both sexes in metabolic studies and the potential for key differences between model species and human disease. Indeed, increasing appreciation of the importance of sexual dimorphism has led the United States National Institutes of Health (NIH) and the UK governmental funding agency (UKRI) (in 2016 and 2022, respectively) to direct researchers to avoid sex-bias in study design [39–41]. Given the great scientific effort spent on understanding the mechanisms behind adipogenesis, lipogenesis and adipocyte browning and the wide usage of murine models to understand these processes, our data demonstrate the importance of performing such studies on both sexes, both *in vitro* and *in vivo*.

## Supporting information

S1 Fig Selected Luminex panel results pg/ml ± SD. Serum collected at end of high fat diet study resulted in 6x Cd248^+/+^ male, 7x Cd248^-/-^ male, 5x Cd248^+/+^ female and 6x Cd248^-/-^ female. Results analysed for significant differences by t test. No significant differences found in either male or female serum.
(TIF)

S2 Fig Lipidomics results for each fat pad. Differences in certain classes of triglycerides (TGs), diglycerides (DGs) alongside Phosphatidylethanolamine (PE) and phosphocholine (PC) are found. **(A)** Heatmap of Cd248^+/+^ compared to Cd248^-/-^, showing differences in lipid classes from male brown adipose tissue. (**B)** Heat map of Cd248^+/+^ and Cd248^-/-^ showing differences in lipid classes from female brown adipose tissue. **(C)** Heat map of Cd248^+/+^ and Cd248^-/-^, showing differences in lipid classes from male inguinal adipose tissue. **(D)** Heat map of CD248^+/+^ and Cd248^-/-^, showing differences in lipid classes from female inguinal adipose tissue. **(E)** Heat map of Cd248^+/+^ and Cd248^-/-^, showing differences in lipid classes from male gonadal adipose tissue. **(F)** Heat map of Cd248^+/+^ and Cd248^-/-^, showing differences in lipid classes from female gonadal adipose tissue. Scale bar from blue = -4 to red = +4 is shown on top left. Lipid notation follows the nomenclature described in Liebisch *et al*. 2013 [22].
(TIF)

S3 Fig Gene expression results each fat pad. These ten genes were found to be significantly altered in the male perirenal fat pad. Their expression in the other fat pads isolated is included here for comparison. No significant differences were observed. Bar graphs showing fold change in expression of ten genes (x axis) in Cd248^-/-^ compared to Cd248^+/+^ mice fat depots: **(A)** male BAT; **(B)** Female BAT; **(C)** Male ingWAT; **(D)** Female ingWAT; **(E)** Male gonWAT and **(F)** Female gonWAT.
(TIF)

S1 Table Lipid information for Fig 4C and 4D. (DOCX)

S2 Table Metabolite information for Fig 5A and 5B. (DOCX)

S3 Table Metabolites information for Fig 5C and 5D. (DOCX)

S4 Table The number of detected metabolites and lipids. (DOCX)

S5 Table OpenArray and Luminex gene/protein list. (DOCX)
