# Supplementary figures and images for: Sex-specific effects of CD248 on metabolism and the adipose tissue lipidome
Source: PLoS One. 2023 Apr 28;18(4):e0284012. doi: 10.1371/journal.pone.0284012 (PMC10146461; doi:10.1371/journal.pone.0284012)

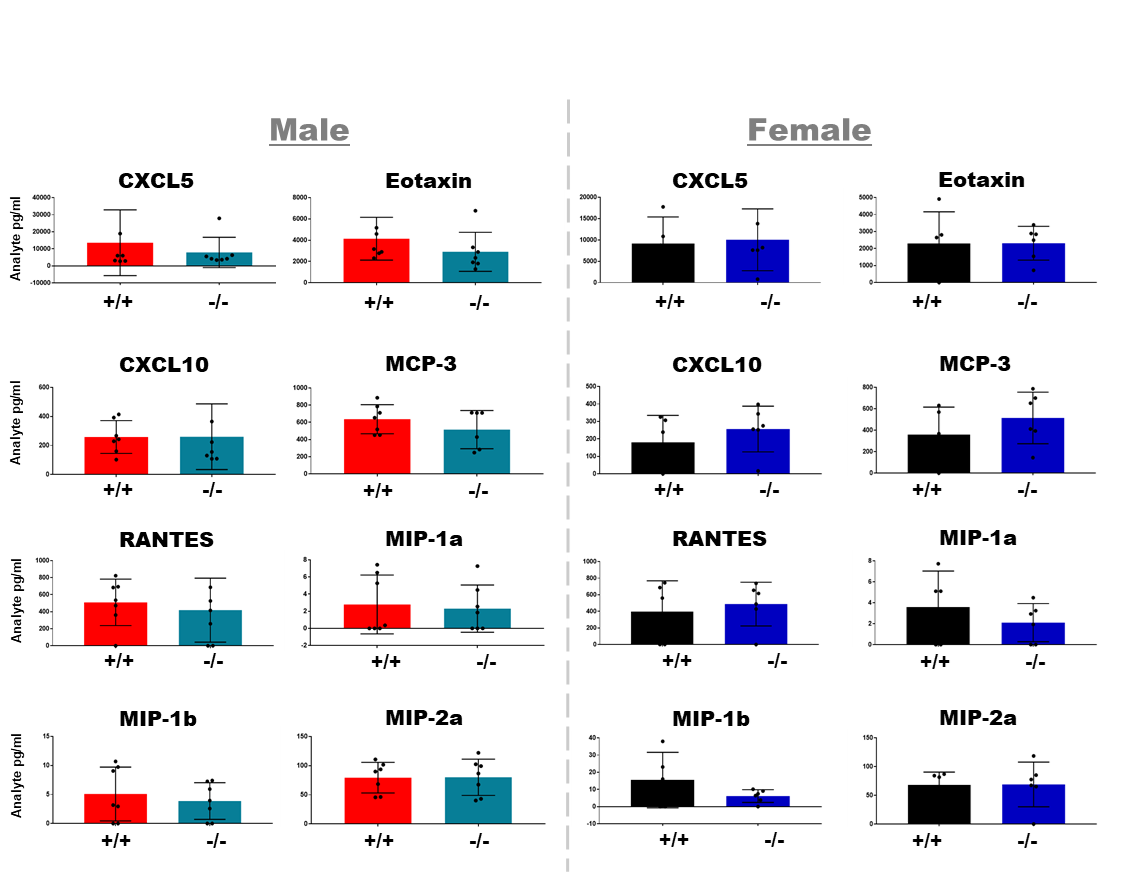

Supplement: S1 Fig — Serum collected at end of high fat diet study resulted in 6x Cd248+/+ male, 7x Cd248-/- male, 5x Cd248+/+ female and 6x Cd248-/- female. Results analysed for significant differences by t test. No significant differences found in either male or female serum. (TIF) [file pone.0284012.s001.tif]

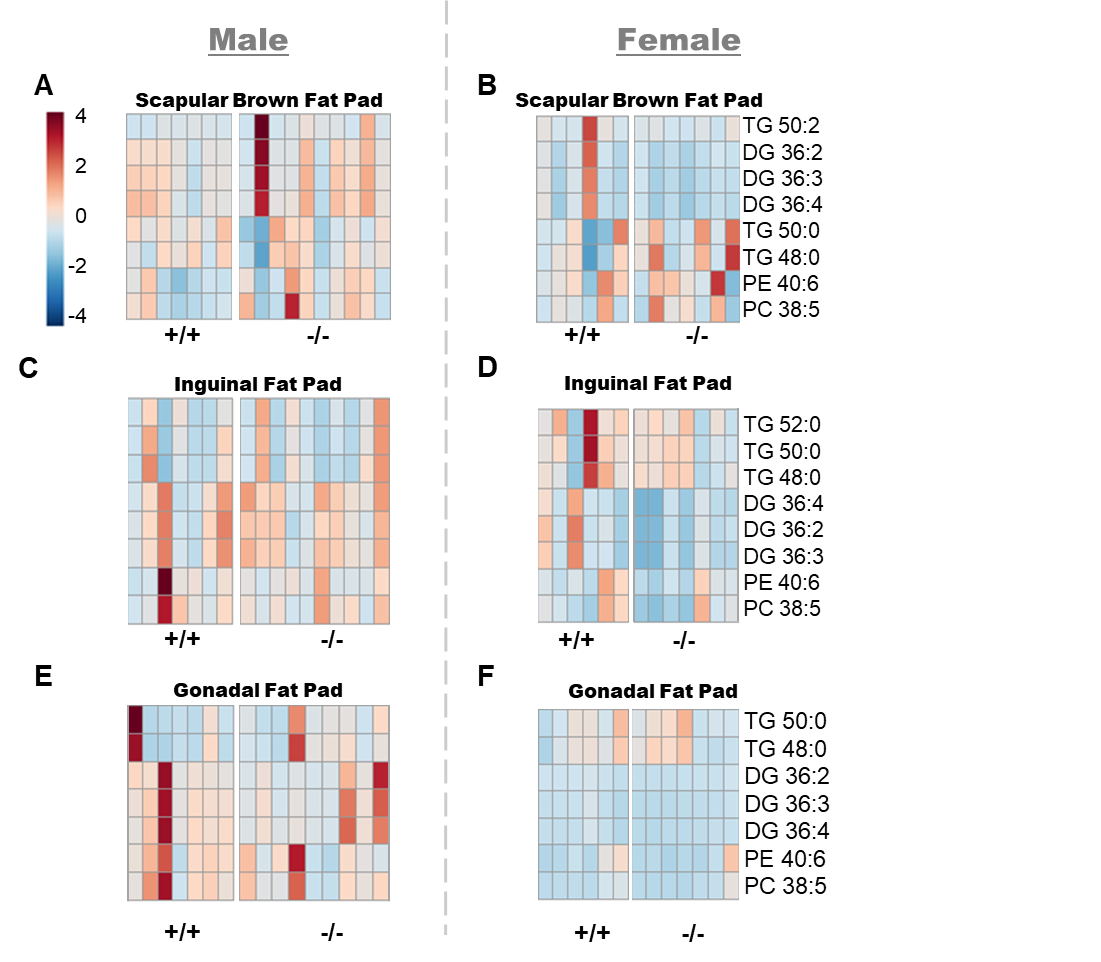

Supplement: S2 Fig — Differences in certain classes of triglycerides (TGs), diglycerides (DGs) alongside Phosphatidylethanolamine (PE) and phosphocholine (PC) are found. (A) Heatmap of Cd248+/+ compared to Cd248-/-, showing differences in lipid classes from male brown adipose tissue. (B) Heat map of Cd248+/+ and Cd248-/- showing differences in lipid classes from female brown adipose tissue. (C) Heat map of Cd248+/+ and Cd248-/-, showing differences in lipid classes from male inguinal adipose tissue. (D) Heat map of CD248+/+ and Cd248-/-, showing differences in lipid classes from female inguinal adipose tissue. (E) Heat map of Cd248+/+ and Cd248-/-, showing differences in lipid classes from male gonadal adipose tissue. (F) Heat map of Cd248+/+ and Cd248-/-, showing differences in lipid classes from female gonadal adipose tissue. Scale bar from blue = -4 to red = +4 is shown on top left. Lipid notation follows the nomenclature described in Liebisch et al. 2013 [22]. (TIF) [file pone.0284012.s002.tif]

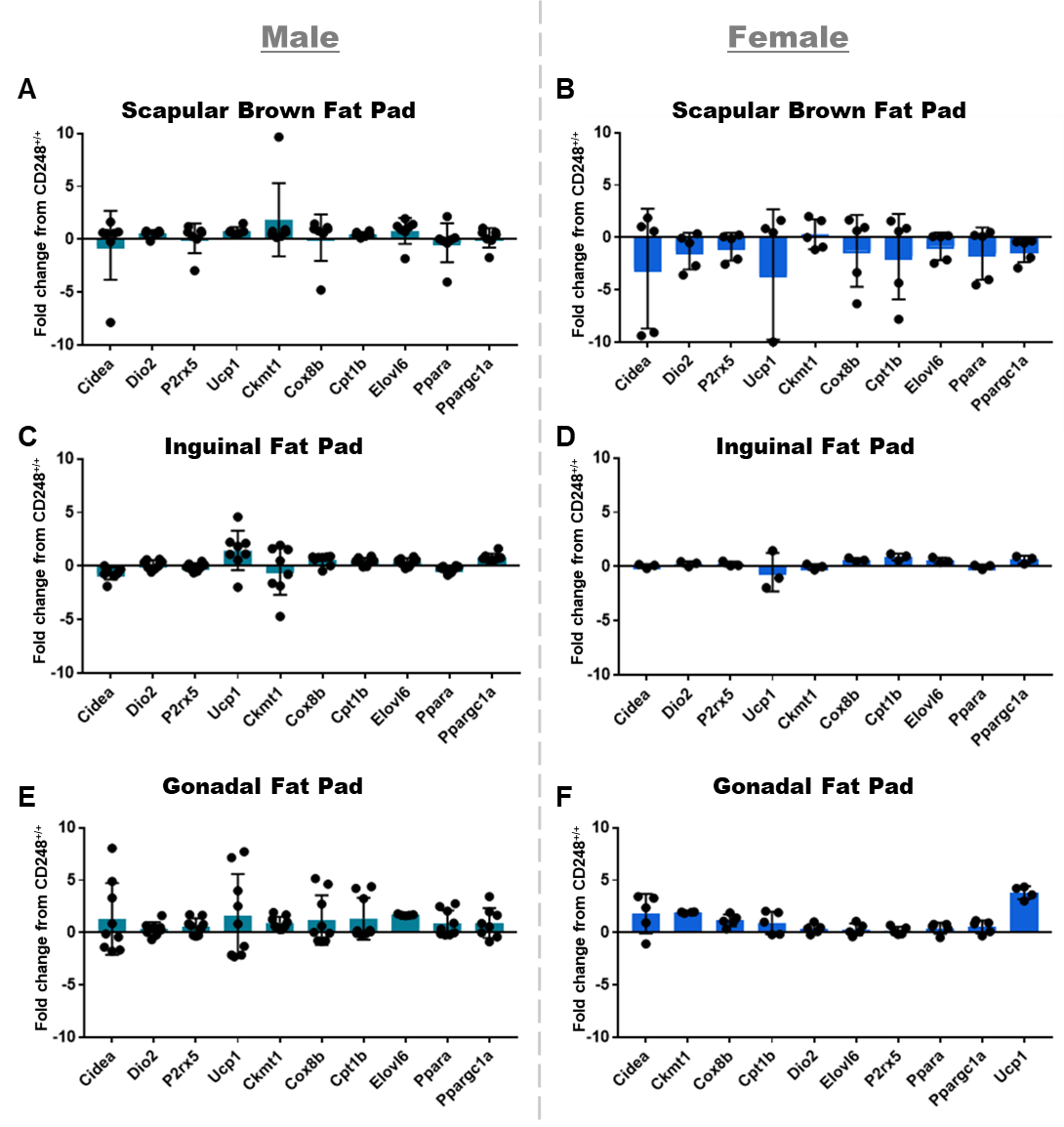

Supplement: S3 Fig — These ten genes were found to be significantly altered in the male perirenal fat pad. Their expression in the other fat pads isolated is included here for comparison. No significant differences were observed. Bar graphs showing fold change in expression of ten genes (x axis) in Cd248-/- compared to Cd248+/+ mice fat depots: (A) male BAT; (B) Female BAT; (C) Male ingWAT; (D) Female ingWAT; (E) Male gonWAT and (F) Female gonWAT. (TIF) [file pone.0284012.s003.tif]
