# Supplementary material for: Sex-specific effects of CD248 on metabolism and the adipose tissue lipidome
Source: PLoS One. 2023 Apr 28;18(4):e0284012. doi: 10.1371/journal.pone.0284012 (PMC10146461; doi:10.1371/journal.pone.0284012)
Supplement: S1 Table — (DOCX) [file pone.0284012.s004.docx]

**Table S1 lipid information for figure 4 C-D**

| **Lipids** | **m/z** | **Adduct** | **MSI Level Identification** |
| --- | --- | --- | --- |
| DG 36:3 | 636.55945 | [M+NH4]+ | Level 2 |
| DG 36:4 | 634.54364 | [M+NH4]+ | Level 2 |
| DG 36:2 | 638.57483 | [M+NH4]+ | Level 2 |
| TG 52:0 | 880.83673 | [M+NH4]+ | Level 2 |
| TG 50:0 | 852.80505 | [M+NH4]+ | Level 2 |
| TG 48:0 | 824.77307 | [M+NH4]+ | Level 2 |
| PE 40:6 | 792.55872 | [M+H]+ | Level 2 |
| PC 38:5 | 808.59009 | [M+H]+ | Level 2 |
