# Supplementary material for: Sex-specific effects of CD248 on metabolism and the adipose tissue lipidome
Source: PLoS One. 2023 Apr 28;18(4):e0284012. doi: 10.1371/journal.pone.0284012 (PMC10146461; doi:10.1371/journal.pone.0284012)
Supplement: S2 Table — (DOCX) [file pone.0284012.s005.docx]

**Table S2 metabolite information for figure 5 A-B**

| **Metabolite** | **m/z** | **Adduct** | **MSI Level Identification** |
| --- | --- | --- | --- |
| Butyryl carnitine | 232.15402 | [M+H]+ | level 2 |
| Carnosine | 227.11359 | [M+H]+ | level 2 |
| Choline | 104.10657 | [M]+ | level 2 |
| Cytosine | 112.05067 | [M+H]+ | level 1 |
| Gluconate | 195.05087 | [M-H]- | level 1 |
| Glutamate | 130.04967 | [M+H-H2O]+ | level 1 |
| Glutarate | 131.03595 | [M-H]- | level 1 |
| Histamine | 112.08696 | [M+H]+ | level 1 |
| Hydroxydecanedioic acid | 217.10825 | [M-H]- | level 1 |
| Hydroxyisobutyrate | 103.03997 | [M-H]- | level 1 |
| Hydroxymethylglutarate | 161.04532 | [M-H]- | level 1 |
| Methionine | 150.05856 | [M+H]+ | level 2 |
| N-acetylalanine | 130.05058 | [M-H]- | level 1 |
| N-acetylgalactosamine | 260.05325 | [M+K]+ | level 1 |
| N-methylglutamate | 162.07513 | [M+H]+ | level 1 |
| Proline-hydroxyproline | 229.11821 | [M+H]+ | level 2 |
| Pyridoxal | 150.05525 | [M+H-H2O]+ | level 1 |
| Pyridoxate | 182.04836 | [M-H]- | level 1 |
| Pyridoxolactone | 166.04982 | [M+H]+ | level 1 |
| Serine | 104.0354 | [M-H]- | level 1 |
| Ureidopropionate | 131.04604 | [M-H]- | level 1 |
