# Supplementary material for: Sex-specific effects of CD248 on metabolism and the adipose tissue lipidome
Source: PLoS One. 2023 Apr 28;18(4):e0284012. doi: 10.1371/journal.pone.0284012 (PMC10146461; doi:10.1371/journal.pone.0284012)
Supplement: S3 Table — (DOCX) [file pone.0284012.s006.docx]

**Table S3. Metabolites information for figure 5 C-D**

| **Name** | **m/z** | **Adduct** | **MSI Level Identification** |
| --- | --- | --- | --- |
| Adenosine | 90.0546 | [M+H]+ | level 1 |
| Alanine | 90.0546 | [M+H]+ | level 2 |
| Arabinose | 195.0505 | [M+FA-H]- | level 1 |
| Carnosine | 227.1141 | [M+H]+ | level 2 |
| Cdp-Ethanolamine | 445.05 | [M-H]- | level 1 |
| Choline | 104.107 | [M]+ | level 2 |
| Creatine | 132.0765 | [M+H]+ | level 2 |
| Deoxyguanosine | 312.0963 | [M+FA-H]- | level 1 |
| Glucosamine | 214.0436 | [M+Cl]- | level 1 |
| Glutamate | 146.042 | [M-H]- | level 1 |
| Glutamine | 147.0765 | [M+H]+ | level 2 |
| Glutarylcarnitine | 276.1441 | [M+H]+ | level 1 |
| Indoxyl Sulfate | 134.0594 | [M+H]+ | level 2 |
| Methylhistamine | 148.0803 | [M+H]+ | level 1 |
| Pantothenic Acid | 134.0594 | [M+H]+ | level 2 |
| Phenylpyruvic Acid | 163.0404 | [M-H]- | level 1 |
| Phosphocholine | 184.0733 | [M]+ | level 2 |
| Propionic Acid | 97.02809 | [M+Na]+ | level 2 |
| Propionylcarnitine | 218.1393 | [M+H]+ | level 2 |
| Pyridoxate | 184.0605 | [M+H]+ | level 2 |
| Pyridoxine | 170.0813 | [M+H]+ | level 1 |
| Pyroglutamic Acid | 130.0499 | [M+H]+ | level 2 |
| Taurine | 124.0051 | [M-H]- | level 2 |
| Thymidine | 241.0829 | [M-H]- | level 1 |
