# Supplementary material for: Sex-specific effects of CD248 on metabolism and the adipose tissue lipidome
Source: PLoS One. 2023 Apr 28;18(4):e0284012. doi: 10.1371/journal.pone.0284012 (PMC10146461; doi:10.1371/journal.pone.0284012)
Supplement: S4 Table — (DOCX) [file pone.0284012.s007.docx]

**Table S4. The number of detected metabolites and lipids**

|  | Lipids | | | | Metabolites | |
| --- | --- | --- | --- | --- | --- | --- |
| Type | gWAT | iWAT | pWAT | sBAT | SVF cells | |
| Polarity | + | + | + | + | + | - |
| Detected features | 4195 | 3601 | 2599 | 2954 | 5306 | 4672 |
| Annotated and filtered | 203 | 191 | 163 | 273 | 124 | 67 |
