# Supplementary material for: Sex-specific effects of CD248 on metabolism and the adipose tissue lipidome
Source: PLoS One. 2023 Apr 28;18(4):e0284012. doi: 10.1371/journal.pone.0284012 (PMC10146461; doi:10.1371/journal.pone.0284012)
Supplement: S5 Table — (DOCX) [file pone.0284012.s008.docx]

**Table S5. OpenArray and Luminex gene/protein list**

| **Target Name OpenArray** | | **Target Name Luminex** |
| --- | --- | --- |
| **Gene name** | **Assay ID** |  |
| Adipoq | Mm00456425_m1 | GM-CSF |
| Adrb3 | Mm02601819_g1 | IFN-gamma |
| Angptl4 | Mm00480431_m1 | IL-1b |
| Atf2 | Mm04409828_s1 | IL-12p70 |
| B2m | Mm00437762_m1 | IL-13 |
| Cav1 | Mm00483057_m1 | IL-18 |
| Cd248 | Mm00547485_s1 | IL-2 |
| CD36 (FAT) | Mm00432403_m1 | IL-4 |
| Cebpa | Mm00514283_s1 | IL-5 |
| Cfd | Mm01143935_g1 | IL-6 |
| Cidea | Mm00432554_m1 | TNF-a |
| Ckmt1 | Mm00438221_m1 | CXCL5 |
| Ckmt2 | Mm01285553_m1 | CSF-3 |
| Col6a3 | Mm00711678_m1 | IFN-a |
| Cox8b | Mm00432648_m1 | IL-1a |
| Cpt1b | Mm00487191_g1 | IL-15 |
| Dio2 | Mm00515664_m1 | IL-28 |
| Elovl6 | Mm00851223_s1 | IL-3 |
| Fasn | Mm00662319_m1 | IL-31 |
| Ffar4 | Mm00725193_m1 | LIF |
| Fgf21 | Mm00840165_g1 | M-CSF |
| Hprt | Mm03024075_m1 | IL-10 |
| Il1b | Mm00434228_m1 | IL-17A |
| Il6 | Mm00446190_m1 | IL-22 |
| Lep | Mm00434759_m1 | IL-23 |
| Lpl | Mm00434764_m1 | IL-27 |
| Mlxipl | Mm02342723_m1 | IL-9 |
| Npr1 | Mm00435309_m1 | Eotaxin |
| Npr3 | Mm00435329_m1 | CXCL1 |
| Nr1h3 | Mm00443451_m1 | CXCL10 |
| Nrf1 | Mm01135606_m1 | MCP-1 |
| Nrip1 | Mm00476537_s1 | MCP-3 |
| P2rx5 | Mm00473677_m1 | MIP-1a |
| Pdgfra | Mm00440701_m1 | MIP-1b |
| Plin1 | Mm00558672_m1 | MIP-2a |
| Pnpla2 | Mm00503040_m1 | RANTES |
| Ppara | Mm00440939_m1 |  |
| Pparg | Mm00440940_m1 |  |
| Ppargc1a | Mm01208835_m1 |  |
| Ppia | Mm02342430_g1 |  |
| Prdm16 | Mm00712556_m1 |  |
| Prkaa2 | Mm01264789_m1 |  |
| Retn | Mm00445641_m1 |  |
| Rplp0 | Mm00725448_s1 |  |
| Slc27a1 | Mm00449511_m1 |  |
| Slc2a4 | Mm00436615_m1 |  |
| Slc36a2 | Mm00462617_m1 |  |
| Slc7a10 | Mm00502045_m1 |  |
| Srebf1 | Mm00550338_m1 |  |
| Tfam | Mm00447485_m1 |  |
| Tmem26 | Mm01173641_m1 |  |
| Tnf | Mm00443258_m1 |  |
| Tnfrsf9 | Mm00441899_m1 |  |
| Ucp1 | Mm01244861_m1 |  |
| Ucp3 | Mm01163394_m1 |  |
| Vegfa | Mm00437306_m1 |  |
